# Use of Phage Cocktail BFC 1.10 in Combination With Ceftazidime-Avibactam in the Treatment of Multidrug-Resistant *Pseudomonas aeruginosa* Femur Osteomyelitis—A Case Report

**DOI:** 10.3389/fmed.2022.851310

**Published:** 2022-04-25

**Authors:** Karlis Racenis, Dace Rezevska, Monta Madelane, Ervins Lavrinovics, Sarah Djebara, Aivars Petersons, Juta Kroica

**Affiliations:** ^1^Department of Biology and Microbiology, Riga Stradins University, Riga, Latvia; ^2^Center of Nephrology, Pauls Stradins Clinical University Hospital, Riga, Latvia; ^3^Joint Laboratory, Pauls Stradins Clinical University Hospital, Riga, Latvia; ^4^Department of Infectology, Riga Stradins University, Riga, Latvia; ^5^Department of Infection Control, Riga East University Hospital, Riga, Latvia; ^6^Latvian Center for Plastic, Reconstructive and Microsurgery, Riga, Latvia; ^7^Centre for Infectious Diseases, Queen Astrid Military Hospital, Brussels, Belgium; ^8^Department of Internal Diseases, Riga Stradins University, Riga, Latvia

**Keywords:** *P. aeruginosa*, biofilm, bacteriophage, phage therapy, osteomyelitis, multidrug resistance

## Abstract

High-energy trauma with severe bone fractures can be complicated by infection, leading to the development of osteomyelitis. *Pseudomonas aeruginosa* is an important causative agent of such infections because of its high virulence profile and ability to develop resistance against a wide range of antimicrobials quickly. *P. aeruginosa* biofilms cause treatment failure and relapsing infections. Bacteriophages are viruses that can be used to treat biofilm-associated infections. Moreover, the combination of phages with certain antimicrobials have demonstrated synergistic and additive effects. We present a case of a 21-year-old patient with relapsing multidrug-resistant (MDR) *P. aeruginosa* femur osteomyelitis that developed after a road accident, with a proximal right femoral Grade III B open fracture and severe soft tissue damage. Despite extensive antimicrobial treatment and multiple surgical interventions with wound debridement, the infection persisted, with subsequent development of femoral osteomyelitis with a fistula. Patient care management included femoral head excision with wound debridement, intravenous (IV) ceftazidime-avibactam, and the local application of the lytic *Pseudomonas* bacteriophage cocktail BFC 1.10. Nine months after the intervention, the patient did not show any clinical, radiological, or laboratory signs of inflammation; therefore, hip replacement was performed. Nevertheless, recurrent *P. aeruginosa* infection evolved at the distal side of the femur and was successfully treated with conventional antimicrobials. In this case, wound debridement combined with antibiotics and bacteriophages resulted in bacterial eradication of proximal femoral segment, avoiding leg amputation, but failed to treat osteomyelitis in distal bone segment. An *in vitro* assessment of the isolated MDR *P. aeruginosa* strain for biofilm formation and phage susceptibility was performed. Additionally, the antimicrobial effects of ceftazidime-avibactam and BFC 1.10 were determined on planktonic cell growth and bacterial biofilm prevention was evaluated. The isolated bacterial strains were susceptible to the bacteriophage cocktail. Strong biofilm formation was detected 6 h after inoculation. Ceftazidime-avibactam combined with BFC 1.10 was most effective in preventing planktonic cell growth and biofilm formation. In both cases, the required concentration of ceftazidime-avibactam decreased two-fold. This study demonstrates the possible use of bacteriophages and antibiotics in difficult-to-treat bone and soft tissue infections, where the additive effects of phages and antibiotics were observed.

## Introduction

Osteomyelitis after severe bone fractures is a well-known complication. The risk of secondary surgical site infection corresponds to the severity of bone fractures according to the Gustilo-Andersson classification (1). Risk factors include biofilm formation and the type of causative agent. *Pseudomonas aeruginosa* is one of the pathogens with the highest recurrence rate owing to its broad antimicrobial resistance, high virulence profile, and the ability to form biofilms, a crucial property for chronic bacterial colonization (2, 3).

The published data illustrate that the prevalence of bacterial biofilms can reach 78.2% in chronic wounds, becoming a formidable challenge in wound care, treatment, and management (4). Multidrug resistance (MDR) and resilient biofilm production are ever-growing challenges in managing *P. aeruginosa* infections. To combat this concern, there is strong interest in developing potentially promising alternatives (5).

Bacteriophage (phage) therapy is a non-antibiotic strategy to circumvent the rise in antibiotic resistance and combat difficult-to-treat infections in clinical settings. Lytic phages cause bacterial cell lysis. Moreover, the activity of their polysaccharide depolymerases helps overcome the carbohydrate boundaries, including extracellular polysaccharides within biofilms (6). These data suggest that bacteriophages might synergize with commonly used antibiotics to eradicate drug-resistant strains; phages might boost the potency of antibiotics (7). Variability in the possible interactions between phages and antibiotics can be determined by the mechanistic action of the chosen antibiotic class (8). Clinical cases have demonstrated the successful use of bacteriophages in bone and soft tissue infection treatment (9–11). It is important to avoid the well-known side effects of the treatment using antimicrobials. Until now, phage therapy has been safe in treating infection; however, the data are limited and may raise concerns in the future (12, 13). Therefore, phages, such as bacteriophage cocktail BFC 1.10, which are well-described and safe for patient care, should be applied for treating infections (14).

We report the case of a 21-year-old man with MDR *P. aeruginosa* osteomyelitis that developed after a trauma-related severe femoral fracture. The patient was treated with conventional antibiotics; however, the infection persisted. Additionally, he developed acute kidney injury, a side effect of colistin treatment. To avoid right leg amputation, surgical intervention with wound debridement and antimicrobial regimen combining IV ceftazidime-avibactam and local lytic *Pseudomonas* bacteriophage cocktail BFC 1.10 was used. We also evaluated biofilm formation by isolated bacterial strain, phage susceptibility, and the impact of phage-antibiotic interactions on planktonic and biofilm-forming cells.

## Case Description

**Figure 1:**
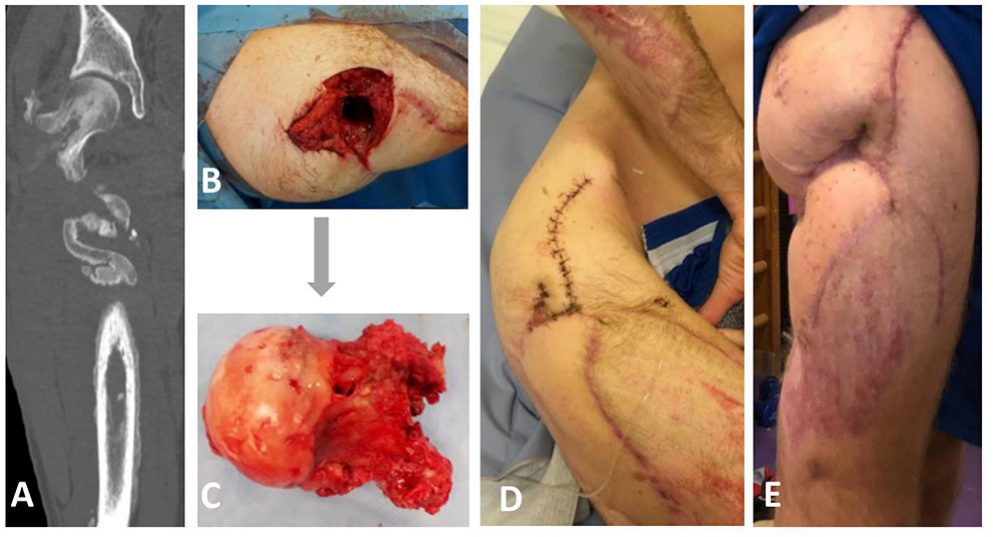
Clinical and radiological appearance of the patient prior, during, and after bacteriophage therapy. **(A)** Computed tomography revealing a fistula that connects femoral head and skin on the right upper third of the lateral femur surface. **(B,C)** Intraoperative findings from the right femoral head excision on the 5th of December revealing the presence of a femoral fistula. **(D)** Postoperative photograph showing irrigation system for local bacteriophage application. **(E)** The appearance of the wound two months after bacteriophage therapy.

In July 2018, a 21-year-old man was hospitalized after a road accident with open comminuted proximal right femoral and acetabular fractures, laceration of the right lower hand, and hemorrhagic shock. Wound debridement, fasciotomy, femur fracture stabilization with gamma nail, and tissue reconstruction were performed on the 14th of July. On consecutive days, the patient developed secondary MDR *P. aeruginosa*, carbapenem-resistant *Acinetobacter baumannii*, and vancomycin-resistant *Enterococcus faecium* (VRE) wound infections and multiple organ dysfunction syndrome. The patient underwent five debridement procedures and therapy using a wound vacuum system. Broad-spectrum IV antimicrobial treatment with meropenem, colistin, piperacillin-tazobactam, linezolid, and fluconazole was administered, and renal replacement therapy was initiated. Regardless of the treatment, the patient developed an osteosynthesis-associated infection and osteomyelitis, and repeatedly positive wound cultures grew with VRE, MDR *P. aeruginosa*, and MDR *A. baumannii*. On the 13th of August, the gamma nail was removed, and proximal femoral segment resection was performed, followed by tissue reconstruction and lower leg external fixation. Based on the antibiogram, the antimicrobial regimen was changed to intravenous fosfomycin, meropenem, and colistin. On the 13th of September, the right thigh wound was closed using a scapular flap. The patient's condition improved gradually, and there were no signs of systemic or local inflammation. Repeated cultures of the wound were negative. The patient was discharged on the 15th of October with IV meropenem and colistin treatment, which was discontinued after two weeks due to acute kidney injury, presumably colistin-induced nephrotoxicity. In November, purulent discharge from the right upper tight appeared. Computed tomography with contrast injection in the cutaneous wound opening revealed a fistula that connects femoral head and skin on the right upper third of the lateral femur surface (Figure 1A). The patient underwent fistulotomy, and MDR *P. aeruginosa* and VRE were isolated from the wound. With a presumptive diagnosis of recurrent femoral osteomyelitis, two-stage surgery was planned to preserve hip replacement surgery in the future. Local bacteriophage therapy was planned using the bacteriophage cocktail BFC 1.10 produced at Queen Astrid Military Hospital in Brussels, Belgium, consisting of phages active against *P. aeruginosa* and *S. aureus*.

## Diagnostic Assessment, Therapeutic Intervention, Follow-Up, and Outcomes

**Figure 2:**
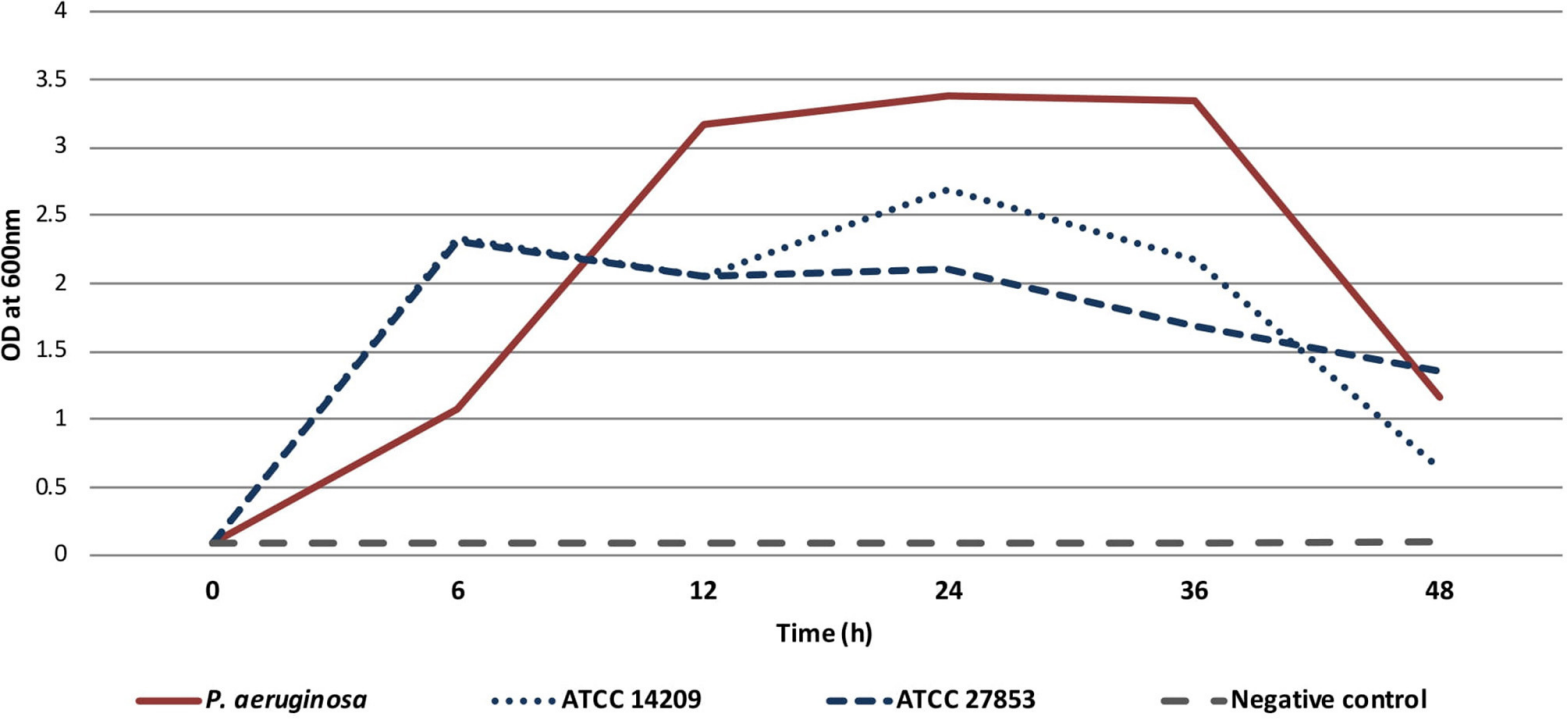
Timeline showing surgical interventions, relevant antimicrobial therapy, and microbiological and pathology findings.

The treatment was performed according to Paragraph 37 of the Declaration of Helsinki (15). The patient provided written informed consent for the use of the bacteriophages. On the 5th of December, a right femoral head excision was performed and replaced with colistin-impregnated cement spacer. The proximal femoral culture was positive for MDR *P. aeruginosa*, VRE, and *Staphylococcus epidermidis*. The pathology and intraoperative findings confirmed femoral head osteomyelitis with fistula (Figures 1B,C). After surgery, the patient was treated with IV colistin for 7 days and linezolid for 23 days. On the 7th of December, 2,000 mL of BFC 1.10 cocktail with 10^7^ plaque-forming units (PFU) per mL of each phage were shipped to Latvia. Prefilled sterile containers containing 30, 40 or 50 mL of phage solution were prepared under sterile conditions. Three days before the procedure, the patient was treated with IV ceftazidime-avibactam, which was continued for 15 days. On the 13th of December, bone cement was removed; wound and acetabular cultures were taken, and were positive for MDR *P. aeruginosa*. Next, wound rinsing with 50 mL BFC 1.10 bacteriophage suspension was performed intraoperatively, tissue damage was replaced with a serratus muscle flap, and an irrigation system for local bacteriophage application was installed (Figure 1D). For the first 7 days, the patient was treated with 40 mL (1 ml/min) of BFC 1.10 three times daily and then with 30 mL (1 ml/min) of BFC 1.10 two times daily via an irrigation catheter for another 7 days. The wound was rinsed with 50 ml of 4.2% sodium bicarbonate solution before the phage application using syringe. Together with the local phage treatment, linezolid and ceftazidime-avibactam were continued. During and after phage treatment on days 1, 3, 4, 7, 10, and 15, no bacterial cultures from the wound grew. Phages were isolated from the wound in the morning buffer sample before phage administration on days 1, 3, 4, 7, 10, and 15. At the end of treatment, the wound healed with no local or systemic signs of infection. When the irrigation catheter was removed, the tip of the catheter was positive for *Candida tropicalis*, which was not treated (Figure 2). No adverse effects, such as fever, local rash, itchiness, or other symptoms, were noted during phage therapy. Patient was discharged with lower leg external fixation until hip replacement surgery. Two months later, the wound healed and there were no signs of inflammation (Figure 1E) that was reassured with magnetic resonance imaging (MRI) of the right hip. Three months after phage treatment, computed tomography of the right hip and femur revealed no fluid collection or signs of osteomyelitis. In the following six months, two punctures from the right femur were performed and were culture negative, three months before hip replacement lower leg external fixation was removed. On the 3rd of September 2019, a hip replacement with a silver-coated implant was performed. During the surgery bacterial cultures were taken, the distal part of the femur was positive for MDR *P. aeruginosa* and VRE (fosfomycin susceptible), but acetabular bone and proximal part muscular tissue were culture negative. Patient received one dose of IV vancomycin for perioperative prophylaxis and IV colistin that was continued until microbiology results. Once the cultures came back positive patient was kept on IV colistin and IV fosfomycin. Sixteen days later DAIR (debridement, antibiotics and implant retention) was performed because of hematoma development and possible prosthesis infection, swabs taken during the surgery from periprosthetic tissue in distal segment were positive for MDR *P. aeruginosa*. On 4th of October punctures from periprosthetic tissue were performed and were culture negative. Three days later patient was discharged and continued antimicrobial therapy in outpatient setting with colistin and fosfomycin. For this episode patient received colistin for six weeks and fosfomycin for five months. During the follow-up period a year later, there were no local signs of infection, and the patient noted limited mobility in the right leg; however, he could continue to play basketball. Radiography of the right hip and femur 15 months later did not reveal any signs of inflammation (Supplementary Figure 1).

## Materials and Methods

### Bacterial Isolate and Reference Strains

The bacterial strains used in this study were as follows: *P. aeruginosa* strain isolated from the patient wound of the right thigh and two *P. aeruginosa* reference strains, ATCC 14209 and ATCC 27853, which served as models for potent biofilm formation ability (16).

### Antimicrobial Susceptibility

To detect antimicrobial susceptibility disk-diffusion test was performed. To detect minimum inhibitory concentration (MIC), the MICRONAUT-S Pseudomonas MIC AST plate by broth microdilution test was used (Merlin-Diagnostika, Germany). Acquired values were interpreted according to the EUCAST.

### Bacteriophage Cocktail

The bacteriophage cocktail BFC 1.10, obtained from the Queen Astrid Military Hospital in Brussels, Belgium, consisted exclusively of lytic *P. aeruginosa* phages 14/1 (exhibiting a myovirus morphotype) and PNM (exhibiting a podovirus morphotype) and *S. aureus* phage ISP (exhibiting a myovirus morphotype). Active pharmaceutical ingredients (API) of the BFC 1, the three phages, were produced in accordance with quality and safety requirements compatible with clinical use. Whole genome sequencing of phages was performed and proved the lytic nature of the phages and the absence of toxin-coding genes. The BFC 1.10 was sterile and purified from the endotoxin, the final pH of the cocktail was 7.29 (14, 17). For local phage application, BFC 1.10 was diluted using 0.9% saline at Queen Astrid Military Hospital to a concentration of 10^7^ PFU/mL for each phage. Safe and potent use of BFC 1.10 has previously been demonstrated in the local and systemic treatment of *P. aeruginosa* and *S. aureus* infections (9, 18, 19). Phage susceptibility was assessed using the spot test on double-layer trypticase soy agar (TSA) plates with BFC 1.10 concentration of 10^7^ PFU/ml for each phage (20).

### Propagation of the Bacteriophage Cocktail BFC 1.10

The propagation procedure was a crucial step in increasing the original titer of the bacteriophage cocktail for *in vitro* testing. The procedures for phage propagation were as follows: selection of webbed plates after plaque assay testing, flooding of the plates with 5–7 ml of trypticase soy broth (TSB), collection of supernatant and soft overlay agar. Afterwards 2% chloroform (CHCl_3_) treatment for 2 h at 4°C, and removal of bacterial debris using centrifugation at 6,000 × *g* for 15 min at 4°C and filtration using a 0.20 μm filter were performed. Phage lysate acquisition was followed by consecutive purification procedure with filter Amicon® Ultra-15 (Merck Millipore Ltd, Ireland) that was executed by centrifugal filtration of 1.5–3 ml previously harvested high titer phage lysate at 4,000 × g for 20 min at 4°C. The supernatant was then collected and the final concentration was determined using plaque assay.

### Model of Biofilm Formation

A sterile 96-well flat-bottomed microtiter plate (96-well TC plate; Suspension, F, Sarstedt, Germany) was used to study biofilm formation. To prepare the bacterial inoculum, three to four morphologically similar colonies from the patient or reference *P. aeruginosa* strain from an overnight culture were selected, inoculated in TSB, and diluted 1:100. Subsequently, 200 μL of the prepared inoculum was pipetted into each well and incubated at 35°C for 6, 12, 24, 36, or 48 h. Sterile TSB was used as a negative control. After incubation, each well was rinsed two times with 250 μL of sterile 0.9% saline. The biofilm was stained with 200 μL of 0.1% crystal violet dye for 25 min, followed by rinsing thrice with 250 μL of distilled water. During decolorization, 200 μL of 96% ethanol was added. Biofilm formation was measured spectrophotometrically at 600 nm (OD_600_) using a 96-well compatible reader (Tecan Infinite F50, Männedorf, Switzerland). A cutoff optical density value of <0.10 was used for bacterial growth.

### BFC 1.10 and Ceftazidime-Avibactam Minimum Inhibitory Concentration (MIC) and Biofilm Prevention Concentration (BPC) Detection

To determine the biofilm formation capacity and the impact of antimicrobials on biofilms, a modified Calgary biofilm method was used. The inoculum was prepared by suspending the colonies of overnight bacterial cultures in TSB. The turbidity of the prepared inoculum was adjusted to 1.0 McFarland standard and diluted to 1:30, reaching a concentration of approximately 1 × 10^7^ CFU/mL. 96-well microplates (Nunc™ MicroWell™ 96-Well, Nunclon Delta-Treated, Flat-Bottom Microplate, Thermo Fisher Scientific, Denmark) containing 150 μL of two-fold diluted antibiotics and/or phages were inoculated with 5 μL of the diluted bacterial suspension and sealed with a 96-peg lid (Nunc™ Immuno TSP Lids). Afterward, the plates were incubated at 35°C for 22 h at 150 rpm. Optical density measurements at OD_650_ were performed to determine the MIC values. The pegged lid was rinsed for two minutes with 200 μL of sterile 0.9% saline and then transferred to a recovery plate containing 200 μL of sterile TSB. Subsequently, the recovery plate was sonicated in an ultrasonication bath (Model 08855-02, Cole-Parmer, USA) for 25 min at a frequency of 44 kHz to dislodge the biofilm. The non-pegged recovery plate was incubated at 35 °C for 22 h, and the optical density was measured at OD_650_ to determine BPC (21–23). BPC values were interpreted and adopted from EUCAST clinical breakpoints.

### Statistical Analysis

IBM SPSS Statistics version 27 and Microsoft Excel 10 were used for data analysis.

## Results

### Bacteriophage Cocktail

Phage spot testing of MDR *P. aeruginosa* isolated from the patient before the treatment showed positive lytic effect with partial lysis that is observed on agar as incomplete lysis of bacteria. Using phage propagation, a high-titer phage stock was obtained, resulting in a significant increase in the phage titer from 1.6 × 10^7^ PFU/mL to 2.5 × 10^9^ PFU/mL.

### Biofilm Formation

**Figure 3:**
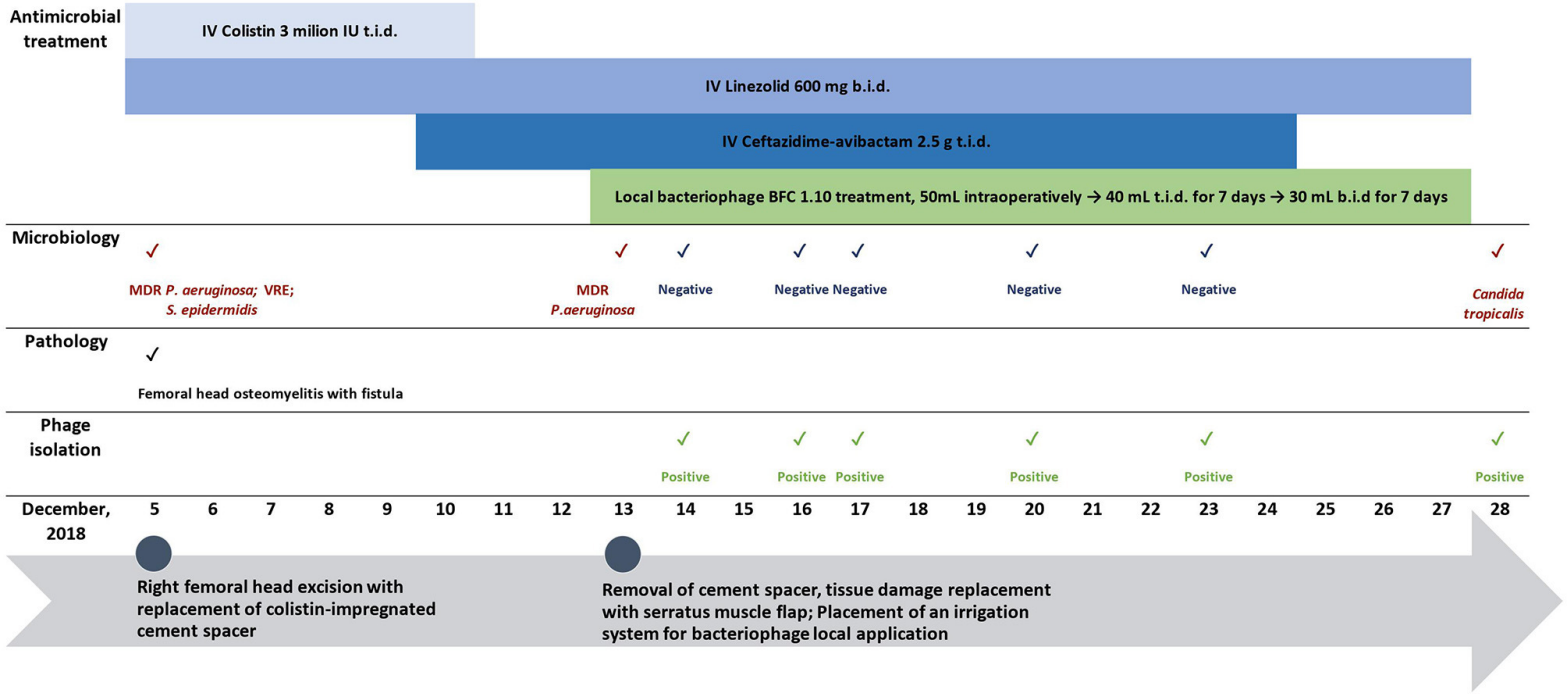
Biofilm formation of isolated *P. aeruginosa* from the patient and two reference strains (ATCC 14209, ATCC 2785) over 48-h. Sterile trypticase soya broth was used as a negative control.

The isolated MDR *P. aeruginosa* strain demonstrated potent (OD_600_ > 1) biofilm formation ability after 6 h, reaching the highest biomass production at 24 h, which persisted until 36 h. Similar results were obtained for both the reference strains (Figure 3).

### The Impact of BFC 1.10 and Ceftazidime-Avibactam on Bacterial Growth and Biofilm Formation

**Figure 4:**
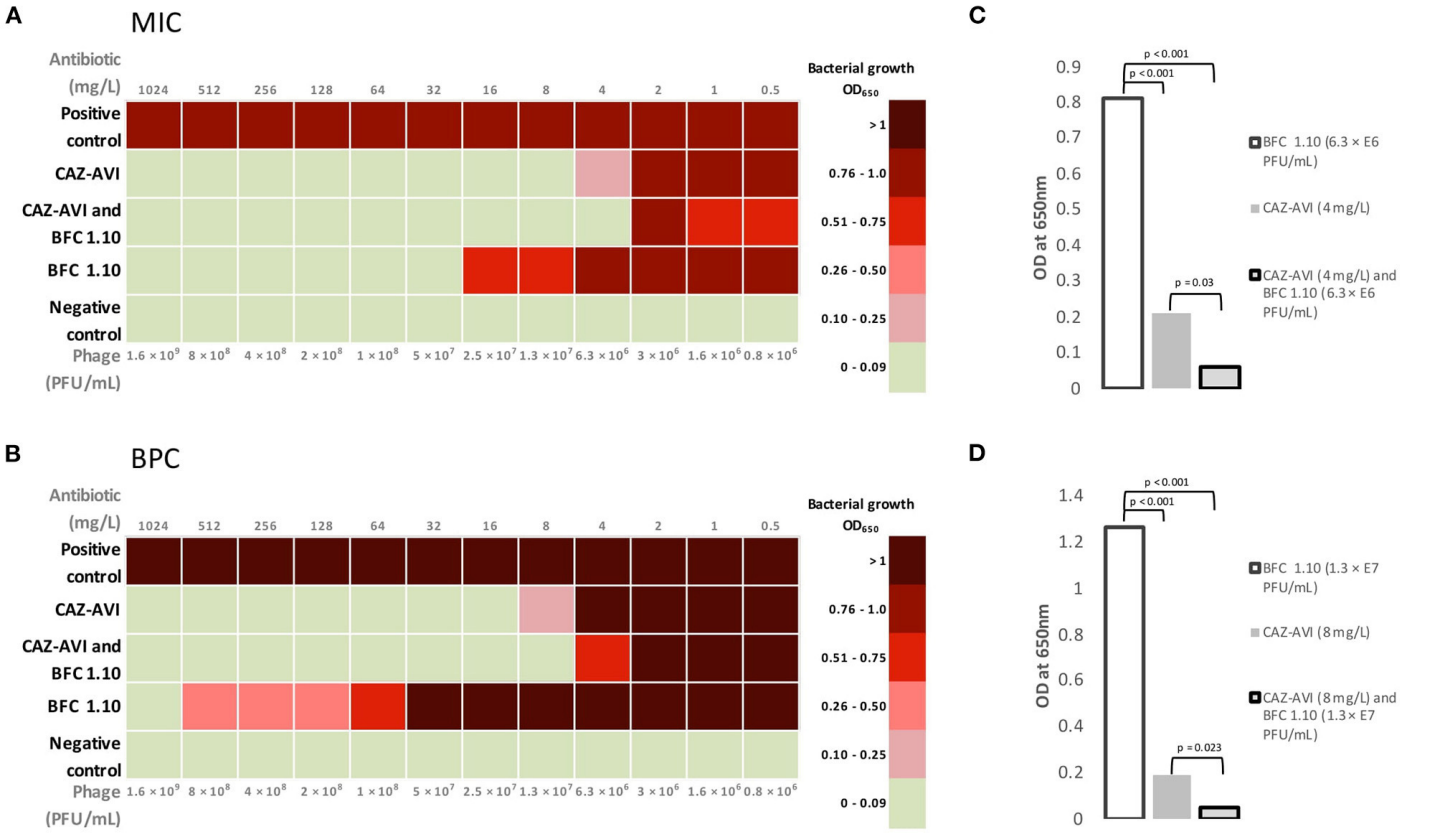
Mean values of minimum inhibitory concentration **(A)** and biofilm prevention concentration **(B)** for BFC 1.10 and/or ceftazidime–avibactam. The graph shows the differences between mean minimum inhibitory concentration **(C)** values using 4 mg/L ceftazidime–avibactam and/or BFC 1.10 6.3 × 10^6^ PFU/mL and mean biofilm prevention concentration **(D)** values using 8 mg/l ceftazidime–avibactam and/or BFC 1.10 3.3 × 10^7^ PFU/mL. Results were analyzed using one-way ANOVA with *post-hoc* Tukey HSD test and were expressed as *p*-values. MIC, minimum inhibitory concentration; BPC, biofilm prevention concentration; CAZ-AVI, ceftazidime-avibactam.

The MIC and BPC values of ceftazidime-avibactam were 8 and 16 mg/L, respectively. Thus, according to the EUCAST standard, acquired values showed susceptibility of planktonic cells but failed to prevent biofilm formation. The phage cocktail BFC 1.10 demonstrated no bacterial growth with a titer of 5 × 10^7^ PFU/mL and prevented biofilm formation only when applied with the highest titer of 1.6 × 10^9^ PFU/mL. An additive antimicrobial effect of ceftazidime-avibactam and BFC 1.10 was observed in the planktonic state of *P. aeruginosa* strain (when detecting MIC) and on prevention of biofilm formation (BPC). When ceftazidime-avibactam was used in combination with BFC 1.10, the MIC and BPC values of ceftazidime-avibactam reduced from 8 to 4 mg/L (*p* = 0.03) and from 16 to 8 mg/L (*p* = 0.023), respectively, compared to those obtained using ceftazidime-avibactam alone (Figure 4). Antibiograms of isolated *P. aeruginosa* strains in different time periods are shown in Supplementary Table 1.

## Discussion

Infections are frequent complications of severe high-energy trauma, and posttraumatic osteomyelitis can develop in up to 19% of cases (24). Antimicrobial resistance has become an emerging major public health problem during the last few decades. Factors such as trauma-related severe bone fractures (Grade III, Gustilo-Anderson classification) and extensive soft tissue damage are associated with a remarkably high risk for developing surgical site infection (SSI) (4–52%) (25). Importantly, a considerable increase in the proportion of infections caused by gram-negative bacilli and polymicrobial flora has been observed, notably in grade III injuries, predicting a poorer prognosis (26). Infections caused by *P. aeruginosa* are difficult to treat and have a high recurrence rate (2, 27, 28). *Pseudomonas* infections require a prolonged treatment course and a combination of two or more different classes of antimicrobials. In cases of osteomyelitis, *P. aeruginosa* infection is associated with an increased risk of amputation. Additionally, *P. aeruginosa* can rapidly reveal a multidrug-resistant profile, making the infection more complicated to manage (29, 30).

Similarly, for the patient in our clinical case, infection was caused by the polymicrobial flora of MDR *P. aeruginosa*, carbapenem-resistant *A. baumannii*, and VRE. In addition to the recurrent infection, femur osteomyelitis, predominantly caused by MDR *P. aeruginosa*, developed despite several debridement procedures and extensive antimicrobial treatment. Multiple factors determine the high virulence and subsequent persistence of infection of *P. aeruginosa*, such as potential adaptation to various environmental factors, production of exotoxins leading to possible severe tissue damage, the rapid development of antimicrobial resistance, and the ability to produce highly structured biofilms (31). The ability *of P. aeruginosa* to produce biofilms is a critical factor that reduces host defense, leading to chronic relapsing infections owing to long-term colonization and bacterial persistence (32). The necrotic tissue and bones in osteomyelitis serve as the surfaces for biofilm-associated infections (33). The presence of biofilms leads to reduced efficacy of the antibiotic treatment due to several factors, such as limited antibiotic diffusion in the biofilm matrix, differences in bacterial metabolic activity, and the quorum sensing system (34). Our study results demonstrate a strong biofilm formation capability of the isolated MDR *P. aeruginosa* (Figure 3) that affected conventional treatment causing failure.

In the case of MDR *P. aeruginosa* infections, potentially life-threatening complications can occur due to the limited treatment options and possible toxic side effects of antimicrobials. Adverse drug effects can complicate further treatment. Drug nephrotoxicity was observed in our patient while using colistin; therefore, the colistin treatment was given with close monitoring and discontinued as soon as possible to preserve renal function.

The isolated MDR *P. aeruginosa* strain was susceptible to ceftazidime-avibactam. However, the risk of resistance development toward ceftazidime-avibactam is high, especially in multidrug-resistant strains (35). Bacteriophages as natural bacterial viruses, cause bacterial lysis and may be used as alternative antimicrobials. Their ability to self-replicate and produce polysaccharide depolymerases makes them an attractive tool for combating *P. aeruginosa* biofilm-associated infections (9). Several *in vitro* studies have shown a synergistic effect between antimicrobials and antipseudomonal bacteriophages; however, such phenomena are not always observed. This may be explained by the mechanism of action of antimicrobials and the difference in the required environmental factors for biofilm formation; the latter can also be a reason for the disparity in laboratory and clinical results (8, 36, 37). Case reports on the management of *P. aeruginosa* periprosthetic joint infection and aortic graft infection have demonstrated the synergistic effect of phages and antibiotics, including ceftazidime, which led to the resolution of biofilm-associated infections (10, 38). Therefore, an antipseudomonal strategy with dual antimicrobial therapy of local bacteriophage application and IV ceftazidime-avibactam combined with surgical intervention and wound debridement was applied. An additive effect of BFC 1.10 and ceftazidime-avibactam for planktonic cell growth and biofilm prevention was observed. Furthermore, the antibiotic concentration required for biofilm prevention decreased to the MIC cutoff value according to the EUCAST standard, making the strain susceptible to ceftazidime-avibactam (Figure 4). Most importantly, our treatment using phages locally and IV antibiotics led to the eradication of infection in proximal part of femur and pelvis, this was crucial for endoprosthesis reconstruction. No less important in terms of patient management, the side effects of applied therapy were not observed.

Six months after completing treatment, the patient's wounds remained dry and closed, and laboratory inflammatory markers remained stable within normal ranges. Despite the resolution of the proximal femur side infection, MDR *P. aeruginosa* and VRE infection persisted at the distal part of femur. This was not anticipated as there were no local or radiological signs of distal part infection. Therefore, our treatment of bacteriophages and antimicrobials did not lead to resolution of infection but led to eradication of osteomyelitis in acetabular bone. Fortunately, distal segment infection after right hip endoprosthesis implantation was successfully treated with DAIR and suppressive therapy using colistin and fosfomycin. To avoid persistent infection in another bone segment more accurate investigation such as labeled leukocyte scintigraphy or PET/CT could be performed. Unfortunately, at that time it was not available in Latvia for osteomyelitis diagnostics. Another solution might be bacteriophage systemic application; however, it is recommended to us phages topically if possible. Phage application in femoral canal could be helpful but, in this case, we did not perform because the canal consisted of sclerotic lesions and it was not possible to insert an irrigation system in it. In case of endoprosthesis associated infections a hydrogel coating with impregnated bacteriophages could be used. Such approach has been described and can retain the implant; however, the data is very limited (39).

## Perspective

We have demonstrated a safe local phage therapy in combination with ceftazidime-avibactam for relapsing biofilm-associated femur osteomyelitis caused by hard-to-treat MDR *P. aeruginosa* that led to eradication of bacterial infection locally where phages were used but failed to treat osteomyelitis in distal bone segment. Future studies are needed to confirm such approaches, ideally through randomized clinical trials. However, several concerns regarding the phage-antibiotic interactions and pharmacodynamics of the phages should also be addressed.

## Data Availability Statement

The raw data supporting the conclusions of this article will be made available by the authors, without undue reservation.

## Ethics Statement

The studies involving human participants were reviewed and approved by Ethics Committee of Riga East Clinical University Hospital. The patients/participants provided their written informed consent to participate in this study.

## Author Contributions

KR wrote the first version of the manuscript. KR, DR, and MM reviewed the literature. MM, EL, KR, and SD planned the patient management. EL and MM participated in patient care. EL performed the surgeries. KR and DR performed laboratory testing and data analysis. KR, DR, JK, and AP conceptualized the manuscript. All authors participated in the improvement of the manuscript, read, and approved the final version of the manuscript.

## Conflict of Interest

The authors declare that the research was conducted in the absence of any commercial or financial relationships that could be construed as a potential conflict of interest.

## Publisher's Note

All claims expressed in this article are solely those of the authors and do not necessarily represent those of their affiliated organizations, or those of the publisher, the editors and the reviewers. Any product that may be evaluated in this article, or claim that may be made by its manufacturer, is not guaranteed or endorsed by the publisher.
