# Supplementary material for: Use of Phage Cocktail BFC 1.10 in Combination With Ceftazidime-Avibactam in the Treatment of Multidrug-Resistant Pseudomonas aeruginosa Femur Osteomyelitis—A Case Report
Source: Front Med (Lausanne). 2022 Apr 25;9:851310. doi: 10.3389/fmed.2022.851310 (PMC9081798; doi:10.3389/fmed.2022.851310)
Supplement: Supplementary file 1 [file Data_Sheet_1.docx]

Supplementary Material

## Supplementary Table

|  | PA (1) | PA (2) | PA (3) | PA (4) | PA (5) | PA (6) | PA (7) |
| --- | --- | --- | --- | --- | --- | --- | --- |
| Isolate description | Wound swab taken during surgery | Tissue sample taken during surgery | Wound swab taken during surgery | Discharge from the fistula | Wound swab from proximal femur taken during surgery | Acetabular swab taken during surgery | Wound swab from distal femur taken during surgery |
| Time of isolation | 18/07/18 | 01/08/18 | 13/08/18 | 23/11/18 | 05/12/18 | 13/12/18 | 03/09/19 |
| Antibiotics |  |  |  |  |  |  |  |
| AMK | R | R | R | R | R  MIC = 48 | R | R |
| FEP | R | N/A | N/A | N/A | N/A | N/A | N/A |
| CAZ | R | R | R | R | R | R | R |
| CAZ/AVI | N/A | N/A | N/A | S  MIC = 4 | S  MIC = 4 | S  MIC = 6 | N/A |
| CIP | R | R | R | R | R | R | R |
| CST | S | S  MIC = 0.75 | S | S | S | S  MIC = 1.5 | S |
| FOF | N/A | MIC = 16 | N/A | MIC = 32 | N/A | N/A | N/A |
| GEN | R | N/A | N/A | R | R | R | R |
| IPM | R | R | R | R | R  MIC > 32 | R | R |
| MEM | R | R | R | N/A | R | R | R |
| TIC | R | N/A | N/A | N/A | N/A | N/A | N/A |
| TIM | R | N/A | N/A | N/A | N/A | N/A | N/A |
| TOB | N/A | R | R | R | R | R | N/A |
| TZP | R | R | R | R | R | R | R |

PA, *Pseudomonas aeruginosa;* N/A, not available; R, resistant; S, susceptible; MIC, minimum inhibitory concentration, mg/l; AMK, amikacin; FEP, cefepime; CAZ, ceftazidime; CAZ/AVI, ceftazidime/avibactam; CIP, ciprofloxacin; CST, colistin; FOF, fosfomycin; GEN, gentamicin; IPM, imipenem; MEM, meropenem; TIC, ticarcillin; TIM, ticarcillin/clavulanate; TOB, tobramycin; TZP, piperacillin/tazobactam.

**Supplementary Table 1.** Antimicrobial susceptibility of clinical *P. aeruginosa* isolates. Interpretation according to EUCAST clinical breakpoints.

## Supplementary Figure

##

**Supplementary Figure 1.** X-Ray of the right thigh 9 days **(A and B)** and fifteen months **(C and D)** after hip replacement with silver-coated implant.
